# Practical application of DENSE in ischemic heart disease

**DOI:** 10.1186/1532-429X-15-S1-P226

**Published:** 2013-01-30

**Authors:** Johan Kihlberg, Henrik Haraldsson, Tino Ebbers, Jan E Engvall

**Affiliations:** 1 CMIV, Linkoping, Sweden; 2 Linköping University, Linköping, Sweden; 3 University of California, San Fransico, San Fransisco, CA, USA

## Background

In clinical practice, perfusion, viability and systolic myocardial function are fundamental to the evaluation of ischemic patients. Deformation of muscle expresses active contraction and can be represented by strain in various directions. Improvements to the "DENSE" technique, "Displacement Encoding with Stimulated Echoes", have shown high accuracy warranting its application to clinical patients. The purpose of this study was to explore potential differences in myocardial deformation determined in patients with various extent of myocardial scar as well as in the corresponding segments of coronary patients without scar.

## Methods

One hundred and twenty patients with a high likelihood of coronary artery disease have been included in this study. As part of their MRI exam (Philips Achieva 1.5T), three short axis (SA) and three longaxis (LA) slices of the left ventricle were obtained for DENSE determination in end-systole. Slice thickness was 6 mm and voxel size 6 x 1.4 x 1.4 mm. In addition, the following MRI sequences were obtained: cine SSFP, adenosine perfusion (SSFP) and late gadolinium enhancement (3D IR-TFE). All measurements were obtained in relation to the AHA seventeen segment model of the left ventricle, excluding the apical cap. DENSE post processing was performed with an in-house developed software calculating radial and when applicable, circumferential or longitudinal strain. Healthy myocardium was determined if scar was absent and wall motion and perfusion were normal (n=18). Scar was defined as any positive gadolinium enhancement (n=25) in the sixteen segments. The segments were aggregated according to their position in relation to the long axis, e.g. base, mid and apex.

## Results

Strain measurements for scar vs non-scar. Segments with scar >50%: SA radial strain -7.36% (5.66) vs non-scar 18.8% (8.76), p<0.0001. SA circumferential strain -3.93%(5.66) versus 8.77%(5.46), p<0.0001. LA longitudinal strain -9.85% (4.26) versus 8.28%(5,06), p=0.017. LA radial strain was 11.3%(9.20) for scar versus 16.9%(8.42) for non-scar, p<0.0001.

## Conclusions

Strain measurements have potential to objectively detect abnormalities in wall deformation not discernible with the human eye. Significant myocardial scar could be identified by strain measurements using DENSE. In this study, the strain values for non-scar hearts were somewhat lower than expected, possibly because they were obtained at end-systole.

## Funding

The Swedish Heart-Lung foundation, the Swedish Research Council och CMIV and the Faculty of Health Sciences.

